# Supplementary material for: Modeling glioblastoma heterogeneity as a dynamic network of cell states
Source: Mol Syst Biol. 2021 Sep 16;17(9):e10105. doi: 10.15252/msb.202010105 (PMC8444284; doi:10.15252/msb.202010105)
Supplement: Supplementary file 6 — Source Data for Figure 5 [file MSB-17-e10105-s004.zip › Figure5A_sourcedata/GSEA_3017/nosigdb_stateC.GseaPreranked.1622795002785/RICHARDS_CAHOY_JNEUROSCI_2008_ASTROCYTE.html]

Details for gene set RICHARDS\_CAHOY\_JNEUROSCI\_2008\_ASTROCYTE[GSEA]

|  || Dataset | state13017 |
| Phenotype | NoPhenotypeAvailable |
| Upregulated in class | na\_neg |
| GeneSet | RICHARDS\_CAHOY\_JNEUROSCI\_2008\_ASTROCYTE |
| Enrichment Score (ES) | -0.3481778 |
| Normalized Enrichment Score (NES) | -2.6235673 |
| Nominal p-value | 0.0 |
| FDR q-value | 0.0 |
| FWER p-Value | 0.0 |
Table: GSEA Results Summary

  

Fig 1: Enrichment plot: RICHARDS\_CAHOY\_JNEUROSCI\_2008\_ASTROCYTE      
 Profile of the Running ES Score & Positions of GeneSet Members on the Rank Ordered List

  

| PROBE | GENE SYMBOL | GENE\_TITLE | RANK IN GENE LIST | RANK METRIC SCORE | RUNNING ES | CORE ENRICHMENT || 1 | MAGI1 |  |  | 6 | 0.652 | -0.0016 | No |
| 2 | RRM2 |  |  | 19 | 0.542 | -0.0185 | No |
| 3 | HMGB2 |  |  | 20 | 0.534 | -0.0090 | No |
| 4 | ZBTB20 |  |  | 30 | 0.482 | -0.0203 | No |
| 5 | GADD45G |  |  | 33 | 0.467 | -0.0164 | No |
| 6 | PBX1 |  |  | 34 | 0.461 | -0.0082 | No |
| 7 | TMPO |  |  | 49 | 0.432 | -0.0314 | No |
| 8 | GAS1 |  |  | 64 | 0.384 | -0.0554 | No |
| 9 | TCF4 |  |  | 65 | 0.384 | -0.0486 | No |
| 10 | SMC4 |  |  | 78 | 0.365 | -0.0685 | No |
| 11 | MPP6 |  |  | 82 | 0.360 | -0.0687 | No |
| 12 | USP1 |  |  | 92 | 0.352 | -0.0823 | No |
| 13 | ASRGL1 |  |  | 93 | 0.350 | -0.0761 | No |
| 14 | NUSAP1 |  |  | 100 | 0.340 | -0.0833 | No |
| 15 | CKB |  |  | 102 | 0.335 | -0.0795 | No |
| 16 | MCM3 |  |  | 114 | 0.324 | -0.0980 | No |
| 17 | PSMC3IP |  |  | 118 | 0.319 | -0.0990 | No |
| 18 | CCDC34 |  |  | 120 | 0.318 | -0.0955 | No |
| 19 | TAGLN3 |  |  | 135 | 0.303 | -0.1210 | No |
| 20 | CCND2 |  |  | 149 | 0.288 | -0.1445 | No |
| 21 | NFIX |  |  | 152 | 0.286 | -0.1438 | No |
| 22 | POLA1 |  |  | 179 | 0.266 | -0.1964 | No |
| 23 | SNCAIP |  |  | 182 | 0.264 | -0.1961 | No |
| 24 | XRCC5 |  |  | 188 | 0.259 | -0.2025 | No |
| 25 | MCM4 |  |  | 189 | 0.259 | -0.1979 | No |
| 26 | PYGB |  |  | 198 | -0.251 | -0.2111 | No |
| 27 | SEC14L2 |  |  | 202 | -0.252 | -0.2132 | No |
| 28 | UPP1 |  |  | 204 | -0.253 | -0.2109 | No |
| 29 | ARHGAP12 |  |  | 207 | -0.254 | -0.2108 | No |
| 30 | HEPACAM |  |  | 216 | -0.257 | -0.2239 | No |
| 31 | TPST2 |  |  | 226 | -0.259 | -0.2391 | No |
| 32 | CLIC1 |  |  | 237 | -0.261 | -0.2565 | No |
| 33 | CCDC6 |  |  | 240 | -0.262 | -0.2562 | No |
| 34 | SAT1 |  |  | 241 | -0.262 | -0.2516 | No |
| 35 | FDFT1 |  |  | 243 | -0.262 | -0.2491 | No |
| 36 | LIMS1 |  |  | 244 | -0.262 | -0.2445 | No |
| 37 | MID1IP1 |  |  | 245 | -0.263 | -0.2398 | No |
| 38 | ZFYVE21 |  |  | 247 | -0.263 | -0.2374 | No |
| 39 | PCGF5 |  |  | 252 | -0.264 | -0.2415 | No |
| 40 | HIVEP3 |  |  | 255 | -0.265 | -0.2412 | No |
| 41 | NPAS3 |  |  | 256 | -0.265 | -0.2365 | No |
| 42 | AGTRAP |  |  | 257 | -0.266 | -0.2318 | No |
| 43 | ID4 |  |  | 261 | -0.267 | -0.2336 | No |
| 44 | AMOT |  |  | 267 | -0.268 | -0.2399 | No |
| 45 | SOCS3 |  |  | 274 | -0.270 | -0.2483 | No |
| 46 | EHD2 |  |  | 275 | -0.270 | -0.2435 | No |
| 47 | DTNA |  |  | 281 | -0.271 | -0.2497 | No |
| 48 | RRBP1 |  |  | 283 | -0.271 | -0.2471 | No |
| 49 | TGFB2 |  |  | 285 | -0.272 | -0.2445 | No |
| 50 | OSMR |  |  | 292 | -0.274 | -0.2528 | No |
| 51 | TSC22D4 |  |  | 303 | -0.279 | -0.2699 | No |
| 52 | SLC1A3 |  |  | 307 | -0.281 | -0.2716 | No |
| 53 | DHCR7 |  |  | 322 | -0.285 | -0.2973 | No |
| 54 | AHR |  |  | 324 | -0.286 | -0.2945 | No |
| 55 | TNIK |  |  | 330 | -0.287 | -0.3004 | No |
| 56 | HEY2 |  |  | 341 | -0.293 | -0.3172 | No |
| 57 | CHST2 |  |  | 350 | -0.297 | -0.3296 | No |
| 58 | MEST |  |  | 352 | -0.299 | -0.3265 | No |
| 59 | TIMP4 |  |  | 354 | -0.300 | -0.3233 | No |
| 60 | ITGB5 |  |  | 356 | -0.300 | -0.3202 | No |
| 61 | UGDH |  |  | 360 | -0.301 | -0.3215 | No |
| 62 | CPEB4 |  |  | 364 | -0.302 | -0.3227 | No |
| 63 | LRP1 |  |  | 366 | -0.304 | -0.3195 | No |
| 64 | PDPN |  |  | 372 | -0.307 | -0.3251 | No |
| 65 | LMNA |  |  | 376 | -0.308 | -0.3262 | No |
| 66 | FOS |  |  | 377 | -0.309 | -0.3207 | No |
| 67 | CSPG5 |  |  | 381 | -0.310 | -0.3218 | No |
| 68 | IGFBP5 |  |  | 384 | -0.311 | -0.3207 | No |
| 69 | DPF3 |  |  | 390 | -0.316 | -0.3261 | No |
| 70 | S100A4 |  |  | 395 | -0.318 | -0.3293 | No |
| 71 | ZFP36L2 |  |  | 401 | -0.321 | -0.3346 | No |
| 72 | CDH4 |  |  | 402 | -0.321 | -0.3289 | No |
| 73 | FGFR3 |  |  | 403 | -0.322 | -0.3232 | No |
| 74 | SLC4A4 |  |  | 415 | -0.332 | -0.3416 | No |
| 75 | LDLR |  |  | 419 | -0.334 | -0.3423 | Yes |
| 76 | MYC |  |  | 421 | -0.336 | -0.3385 | Yes |
| 77 | S100A6 |  |  | 423 | -0.336 | -0.3347 | Yes |
| 78 | RHOQ |  |  | 426 | -0.338 | -0.3331 | Yes |
| 79 | METRN |  |  | 430 | -0.340 | -0.3337 | Yes |
| 80 | PPP2R2B |  |  | 435 | -0.344 | -0.3364 | Yes |
| 81 | VEGFA |  |  | 436 | -0.345 | -0.3303 | Yes |
| 82 | WWTR1 |  |  | 437 | -0.345 | -0.3242 | Yes |
| 83 | KCNIP1 |  |  | 440 | -0.346 | -0.3225 | Yes |
| 84 | FAT1 |  |  | 441 | -0.346 | -0.3163 | Yes |
| 85 | HSPA5 |  |  | 447 | -0.350 | -0.3211 | Yes |
| 86 | BCAN |  |  | 449 | -0.351 | -0.3171 | Yes |
| 87 | SERPINE2 |  |  | 452 | -0.355 | -0.3152 | Yes |
| 88 | NEK6 |  |  | 453 | -0.355 | -0.3089 | Yes |
| 89 | DKK3 |  |  | 454 | -0.356 | -0.3026 | Yes |
| 90 | RHOC |  |  | 455 | -0.359 | -0.2962 | Yes |
| 91 | SEPT9 |  |  | 457 | -0.361 | -0.2920 | Yes |
| 92 | EDNRB |  |  | 462 | -0.367 | -0.2943 | Yes |
| 93 | NTRK3 |  |  | 464 | -0.368 | -0.2900 | Yes |
| 94 | PDLIM4 |  |  | 466 | -0.370 | -0.2856 | Yes |
| 95 | ALCAM |  |  | 467 | -0.370 | -0.2791 | Yes |
| 96 | B2M |  |  | 468 | -0.371 | -0.2725 | Yes |
| 97 | HES1 |  |  | 470 | -0.373 | -0.2681 | Yes |
| 98 | NQO1 |  |  | 479 | -0.381 | -0.2789 | Yes |
| 99 | HBEGF |  |  | 480 | -0.386 | -0.2721 | Yes |
| 100 | RAB13 |  |  | 482 | -0.388 | -0.2674 | Yes |
| 101 | IFITM2 |  |  | 483 | -0.388 | -0.2605 | Yes |
| 102 | VCL |  |  | 484 | -0.388 | -0.2536 | Yes |
| 103 | AP1S2 |  |  | 489 | -0.395 | -0.2554 | Yes |
| 104 | COTL1 |  |  | 494 | -0.409 | -0.2570 | Yes |
| 105 | FABP7 |  |  | 495 | -0.411 | -0.2497 | Yes |
| 106 | AGT |  |  | 497 | -0.412 | -0.2446 | Yes |
| 107 | FJX1 |  |  | 501 | -0.418 | -0.2438 | Yes |
| 108 | JUN |  |  | 503 | -0.425 | -0.2384 | Yes |
| 109 | F3 |  |  | 505 | -0.432 | -0.2330 | Yes |
| 110 | SLC25A18 |  |  | 507 | -0.433 | -0.2275 | Yes |
| 111 | SCRG1 |  |  | 508 | -0.434 | -0.2198 | Yes |
| 112 | DBI |  |  | 514 | -0.440 | -0.2230 | Yes |
| 113 | PTN |  |  | 517 | -0.446 | -0.2195 | Yes |
| 114 | CA2 |  |  | 518 | -0.448 | -0.2115 | Yes |
| 115 | CST3 |  |  | 520 | -0.452 | -0.2057 | Yes |
| 116 | CEBPD |  |  | 523 | -0.455 | -0.2020 | Yes |
| 117 | HMGCS1 |  |  | 529 | -0.466 | -0.2048 | Yes |
| 118 | CTSC |  |  | 531 | -0.470 | -0.1986 | Yes |
| 119 | IFITM3 |  |  | 533 | -0.477 | -0.1924 | Yes |
| 120 | ITGA6 |  |  | 535 | -0.479 | -0.1861 | Yes |
| 121 | TTYH1 |  |  | 540 | -0.496 | -0.1861 | Yes |
| 122 | SEMA6D |  |  | 541 | -0.500 | -0.1772 | Yes |
| 123 | RAMP1 |  |  | 548 | -0.517 | -0.1813 | Yes |
| 124 | GJA1 |  |  | 552 | -0.527 | -0.1785 | Yes |
| 125 | TIMP3 |  |  | 553 | -0.530 | -0.1691 | Yes |
| 126 | GDF15 |  |  | 554 | -0.531 | -0.1597 | Yes |
| 127 | SPARCL1 |  |  | 557 | -0.544 | -0.1544 | Yes |
| 128 | ANXA2 |  |  | 563 | -0.567 | -0.1554 | Yes |
| 129 | TNC |  |  | 564 | -0.572 | -0.1452 | Yes |
| 130 | PCDH10 |  |  | 565 | -0.573 | -0.1351 | Yes |
| 131 | SPARC |  |  | 567 | -0.575 | -0.1271 | Yes |
| 132 | S100A16 |  |  | 569 | -0.582 | -0.1189 | Yes |
| 133 | FGFBP3 |  |  | 573 | -0.644 | -0.1141 | Yes |
| 134 | GFAP |  |  | 574 | -0.659 | -0.1024 | Yes |
| 135 | CD44 |  |  | 575 | -0.659 | -0.0907 | Yes |
| 136 | VIM |  |  | 578 | -0.674 | -0.0832 | Yes |
| 137 | CLU |  |  | 581 | -0.690 | -0.0753 | Yes |
| 138 | CYR61 |  |  | 584 | -0.714 | -0.0671 | Yes |
| 139 | ATP1B2 |  |  | 585 | -0.714 | -0.0544 | Yes |
| 140 | EMP1 |  |  | 589 | -0.785 | -0.0471 | Yes |
| 141 | PMP22 |  |  | 590 | -0.837 | -0.0322 | Yes |
| 142 | MT3 |  |  | 591 | -0.837 | -0.0174 | Yes |
| 143 | FABP5 |  |  | 596 | -1.477 | -0.0000 | Yes |
Table: GSEA details [plain text format]

  

Fig 2: RICHARDS\_CAHOY\_JNEUROSCI\_2008\_ASTROCYTE: Random ES distribution      
 Gene set null distribution of ES for **RICHARDS\_CAHOY\_JNEUROSCI\_2008\_ASTROCYTE**
